# Hypertension and risk factors in Peruvian outpatients

**DOI:** 10.15649/cuidarte.5177

**Published:** 2026-06-12

**Authors:** Lizbeth Cieza Najarro, Carmencita Muñoz Estela, José Ander Asenjo-Alarcón

**Affiliations:** 1 Registered Nurse. Universidad Nacional Autónoma de Chota. Cajamarca, Peru. E-mail: lis97najarro@gmail.com Universidad Nacional Autónoma de Chota Cajamarca Peru lis97najarro@gmail.com; 2 Registered Nurse. Universidad Nacional Autónoma de Chota. Cajamarca, Peru. E-mail: karmenestela@gmail.com Universidad Nacional Autónoma de Chota Cajamarca Peru karmenestela@gmail.com; 3 PhD in Health Sciences, Epidemiologist. Faculty of Health Sciences, Universidad Nacional Autónoma de Chota. Cajamarca, Peru. E-mail: ander1213@hotmail.com Universidad Nacional Autónoma de Chota Cajamarca Peru ander1213@hotmail.com

**Keywords:** Chronic Disease, Hypertension, Risk Factors, Health Centers, Primary Health Care, Peru, Enfermedad Crónica, Hipertensión, Factores de Riesgo, Centros de Salud, Atención Primaria de Salud, Perú, Doença Crónica, Hipertensão, Fatores de Risco, Centros de Saúde, Atenção Primária à Saúde, Peru

## Abstract

**Introduction:**

Hypertension has multiple risk factors that need to be identified in specific contexts to guide the prioritization and implementation of effective control strategies.

**Objective:**

To assess the association between hypertension and risk factors in Peruvian outpatients.

**Materials and Methods:**

An analytical, retrospective, observational study was conducted in 286 patients attending a primary healthcare center. High blood pressure and risk factor values were obtained using two diagnostic interpretation guides adapted by official institutions. Univariate analysis was performed using frequency distributions, and associations were assessed with the chi-square test for homogeneity (p<0.05) and prevalence odds ratios.

**Results:**

A total of 37.76% of patients had hypertension. The most frequent risk factors were low HDL cholesterol (56.29%) and elevated body mass index (60.49%). Elevated HbA1c was associated with a more than threefold increased risk of hypertension. Elevated total cholesterol, LDL cholesterol, and fasting blood glucose were associated with a more than twofold increased risk in women. In men, increased waist circumference was associated with a more than threefold increased risk, and in older adults, with a more than twofold increased risk.

**Discussion:**

Context shapes the association between hypertension and risk factors, given the specific characteristics of each setting and differing social determinants.

**Conclusion:**

Hypertension was significantly associated with elevated HbA1c, total cholesterol, LDL cholesterol, fasting blood glucose, and waist circumference. Timely and appropriate interventions targeting the identified risk factors could slow the occurrence of hypertension.

## Resumen

**Introducción:**

La hipertensión arterial presenta factores de riesgo, que requieren ser identificados en contextos específicos para la priorización y operativización de estrategias de control efectivas.

**Objetivo:**

Asociar la hipertensión arterial y los factores de riesgo en pacientes ambulatorios peruanos.

**Materiales y métodos:**

Estudio analítico, retrospectivo y observacional, efectuado con 286 pacientes que acudieron a un centro de salud primario. Los valores de hipertensión arterial y factores de riesgo se obtuvieron mediante dos guías de interpretación diagnóstica, adaptadas de instituciones oficiales. El análisis univariado fue a través de frecuencias y la asociación con el chi cuadrado de homogeneidad (p<0,05) y odds ratio de prevalencia.

**Resultados:**

El 37,76% de pacientes tenían hipertensión arterial, los factores de riesgo más frecuentes fueron el C-HDL disminuido (56,29%) y el índice de masa corporal elevado (60,49%), la HbA1c elevada representó más de 3 veces riesgo para hipertensión arterial, el C-Total, el C-LDL y la glucemia en ayunas elevados más de 2 veces riesgo en mujeres, en varones el perímetro abdominal elevado más de 3 veces riesgo y en adultos mayores más de 2 veces riesgo.

**Discusión:**

El contexto determina la asociación de la hipertensión arterial con los factores de riesgo, debido a las características propias de cada lugar y a los determinantes sociales diferenciados.

**Conclusiones:**

La hipertensión arterial se asoció de manera significativa con la HbA1c, C-Total, C-LDL, glucemia en ayunas y perímetro abdominal elevados. La intervención oportuna y acertada sobre los factores de riesgo identificados, ralentizaría la ocurrencia de hipertensión arterial.

## Resumo

**Introdução:**

A hipertensão apresenta fatores de risco que precisam ser identificados em contextos específicos para a priorização e implementação de estratégias de controle eficazes.

**Objetivo:**

Determinar a associação entre hipertensão e fatores de risco em pacientes ambulatoriais peruanos.

**Materiais e métodos:**

Trata-se de um estudo analítico, retrospectivo e observacional realizado com 286 pacientes atendidos em um centro de atenção primária à saúde. Os valores de hipertensão e fatores de risco foram obtidos utilizando dois guias de interpretação diagnóstica adaptados de instituições oficiais. A análise univariada foi realizada utilizando frequências, e as associações foram avaliadas pelo teste qui-quadrado para homogeneidade (p<0,05) e pela razão de chances para prevalência.

**Resultados:**

37,76% dos pacientes apresentavam hipertensão. Os fatores de risco mais frequentes foram colesterol HDL reduzido (56,29%) e índice de massa corporal elevado (60,49%). A hemoglobina glicada (HbA1c) elevada representou um risco mais de três vezes maior para hipertensão. Níveis elevados de colesterol total, colesterol LDL e glicemia de jejum representaram um risco mais que duas vezes maior em mulheres, enquanto circunferência abdominal elevada representou um risco mais que três vezes maior em homens e mais que duas vezes maior em idosos.

**Discussão:**

O contexto determina a associação da hipertensão com os fatores de risco, devido às características específicas de cada local e aos determinantes sociais diferenciados.

**Conclusões:**

A hipertensão foi significativamente associada a níveis elevados de HbA1c, colesterol total, colesterol LDL, glicemia de jejum e circunferência abdominal. Intervenções oportunas e adequadas sobre os fatores de risco identificados podem retardar o desenvolvimento da hipertensão.

## Introduction

As a chronic condition, hypertension is often diagnosed after years of disease progression in patients. Its severity is determined not only by the underlying disease process but also by its complications, which make it the leading cardiovascular risk factor and, consequently, increase cardiovascular morbidity and mortality^1^. Worldwide, more than one-quarter of the population has hypertension, and over half of affected individuals do not achieve adequate disease control. This results in a range of cardiovascular, ocular, renal, and cognitive complications that collectively increase the global burden of disease^2^.

Hypertension has been associated with modifiable risk factors, including excess body weight, which affects nearly half (47.90%) of the Asian population, particularly in urban settings. This is largely attributable to industrialization processes that promote the consumption of packaged foods, reduced physical activity, and, consequently, alterations in biochemical markers^3^. One such marker is high-density lipoprotein cholesterol (HDL cholesterol), which is decreased in patients with hypertension and is inversely associated with increases in blood pressure and other lipid fractions, such as low-density lipoprotein cholesterol (LDL cholesterol)^4^. These findings reflect dietary imbalances and represent a cascade of functional alterations throughout the course of the disease. In this regard, a study conducted in China identified higher body mass index (BMI), total cholesterol, LDL cholesterol, and fasting blood glucose as factors associated with hypertension, particularly in women^5^.

Elevated glycated hemoglobin (HbA1c), as an intermediate-term indicator, increases the risk of hypertension in the early stages of diagnosis^6^. This marker is also associated with type 2 diabetes mellitus, suggesting a shared etiopathogenesis between both conditions and an interrelated trajectory in disease progression and the development of complications. Likewise, fasting blood glucose elevation coexists with hypertension, and both synergistically exacerbate cardiovascular damage in patients, increasing healthcare utilization and both direct and indirect healthcare costs. It remains unclear whether hypertension precedes diabetes or vice versa; however, effective blood pressure control increases the likelihood of achieving glycemic control in patients^7^.

Increased waist circumference, or central obesity, is also an important risk factor for hypertension. As abdominal circumference increases, blood pressure (mmHg) rises proportionally, and this occurs from the early stages of the disease. Central obesity is associated with the accumulation of abdominal fat deposits, driven by alterations in lipid and carbohydrate metabolism^8^. In addition, central obesity disrupts the normal functioning of the immune and endocrine systems and increases the risk of insulin resistance, elevated blood pressure, diabetes, and cardiovascular disease^9^.

To varying degrees, risk factors contribute to the development of hypertension through complex and multifactorial mechanisms; therefore, interventions should be tailored to each specific context to achieve appropriate outcomes^10^. In this regard, characterizing each patient’s risk profile based on internal and external factors facilitates the design of preventive strategies to modify, eliminate, or slow the progression of risk factors and the onset or progression of hypertension. As a noncommunicable disease, hypertension is amenable to prevention and control.

Understanding the characteristics of each risk factor and its association with hypertension within the specific context in which patients or vulnerable populations reside enables more targeted, context-specific actions to raise awareness of the medium- and long-term harms of prolonged exposure to certain risk factors. Active participation of the population is therefore essential to control this situation. To make this association evident, the objective of this study was to assess the association between hypertension and risk factors in Peruvian outpatients.

## Materials and Methods

An analytical, retrospective, observational study was conducted between February and March 2022 in 286 patients attending the Patrona de Chota Health Center in Peru. The study population included patients aged 30 years and older, of both sexes, who were registered and had received care through the end of December 2021, and for whom sufficient information was available in their medical records. Patients with missing data required for the study were excluded.

Data from medical records were collected using document analysis. Data extraction was performed using two diagnostic assessment instruments: the first, adapted from the American Heart Association^11^, was used to evaluate hypertension (normal blood pressure: systolic blood pressure [SBP] <120 mmHg and diastolic blood pressure [DBP] <80 mmHg; elevated: SBP 120–129 mmHg and DBP <80 mmHg; hypertension stage 1: SBP 130–139 mmHg or DBP 80–89 mmHg; hypertension stage 2: SBP ≥140 mmHg or DBP ≥90 mmHg; hypertensive crisis: SBP ≥180 mmHg and DBP >120 mmHg); and the second, adapted from the Ministry of Health of Peru^12^, was used to assess risk factors (smoking: yes/no; total cholesterol: 125–199 mg/dL [normal], ≥200 mg/dL [elevated]; LDL cholesterol: <130 mg/ dL [normal], ≥130 mg/dL [elevated]; HDL cholesterol: men >40 mg/dL [normal], ≤40 mg/dL [low], and women >50 mg/dL [normal], ≤50 mg/dL [low]; triglycerides: <150 mg/dL [normal], ≥150 mg/dL [elevated]; fasting blood glucose: 100–125 mg/dL [normal], ≥126 mg/dL [elevated]; HbA1c: ≤8.3% [normal], >8.3% [elevated]; body mass index [BMI]: <25 kg/m² [normal], 25–29.9 kg/m² [overweight], and ≥30 kg/m² [obesity]; waist circumference: men <102 cm [normal], ≥102 cm [increased], and women <88 cm [normal], ≥88 cm [increased]). Normal and abnormal values were coded to assess associations between variables.

For data collection, permission and formal authorization were obtained from the director of the health center where the study was conducted. Following approval, access to medical records was arranged with the head of the admissions unit from February to March 2022, during daytime hours. Once the relevant records were identified, data were extracted and transcribed into the diagnostic assessment instruments, according to the inclusion criteria, for subsequent analysis.

For univariate analysis, absolute and relative frequencies were calculated, along with 95% confidence intervals. Associations between variables were assessed using the chi-square test of homogeneity (p<0.05) and prevalence odds ratios, with stratified analyses by sex and age using the Mantel–Haenszel test. Statistical analyses were performed using SPSS version 26. The data are available in Mendeley Data^13^.

The study adhered to ethical principles. Data were collected from medical records following written authorization from the director of the Patrona de Chota Health Center and the head of the admissions unit of the same institution. The study was also approved by a scientific committee of the Faculty of Health Sciences at the Universidad Nacional Autónoma de Chota, Peru (Faculty Resolution No. 027-2022-FCCSS-UNACH/C). Data were handled exclusively by the researchers, ensuring confidentiality and discretion required for research involving human participants.

## Results

A considerable proportion of patients had hypertension (37.76%) Table 1; of these, more than half were older adults (66.67%) and women (67.59%), with a median disease duration of 4.50 years [Q1: 2–Q3: 7].

**Table 1:** Frequency of hypertension in patients attending a Peruvian health center. (n = 286)

Hypertension status	% (n)	95% CI
Present	37.76 (108)	32.14 – 43.38
Absent	62.24 (178)	56.62 – 67.86

CI: Confidence interval

The most frequent risk factors were low HDL cholesterol (56.29%) and elevated BMI (60.49%), which may reflect high intake of carbohydrates, saturated fats, or trans fats Table 2.

**Table 2:** Risk factors for hypertension in patients attending a Peruvian health center. (n=286)

Risk factors	% (n)	95% CI
Smoking		
Yes	10.14 (29)	6.64 - 13.64
No	89.86 (257)	86.36 - 93.36
Total cholesterol (mg/dL)		
Elevated (≥200)	22.03 (63)	17.23 - 26.83
Normal	78.97 (223)	74.25 - 83.69
LDL cholesterol (mg/dL)		
Elevated (≥130)	16.08 (46)	11.82 - 20.34
Normal	83.92 (240)	79.66 - 88.18
HDL cholesterol (mg/dL)		
Low (men: <41, women: <51)	56.29 (161)	50.54 - 62.04
Normal	43.71 (125)	37.96 - 49.46
Triglycerides (mg/dL)		
Elevated (≥150)	34.27 (98)	28.77 - 39.77
Normal	65.73 (188)	60.23 - 71.23
Fasting blood glucose (mg/dL)		
Elevated (≥126)	9.79 (28)	6.35 - 13.23
Normal	90.21 (258)	86.77 - 93.65
HbA1c (%)		
Elevated (>8.3)	4.89 (14)	2.39 - 7.39
Normal	95.11 (272)	92.61 - 97.61
BMI (kg/m²)		
Obesity (≥30)	19.93 (57)	15.30 - 24.56
Overweight (25–29.9)	40.56 (116)	34.87 - 46.25
Normal	39.51 (113)	33.84 - 45.18
Waist circumference (cm)	39.51 (113)	33.84 - 45.18
Increased (men: ≥102, women: ≥88)	47.90 (137)	42.11 - 53.69
Normal	52.10 (149)	46.31 - 57.89

CI: Confidence interval; LDL: low-density lipoprotein; HDL: high-density lipoprotein; HbA1c: glycated hemoglobin; BMI: body mass index.

Elevated HbA1c was associated with a 3.14-fold increased risk of hypertension. Elevated total cholesterol, LDL cholesterol, and fasting blood glucose were associated with more than a twofold increased risk in women. In men, increased waist circumference was associated with a 3.28-fold increased risk, and in older adults, with a 2.43-fold increased risk of the disease. These associations were statistically significant (p<0.05); full results are presented in Table 3.

**Table 3:** Association between hypertension and risk factors in patients attending a Peruvian health center

Risk factors	HTN % (n)	No HTN % (n)	PR OR (95% CI)	Adjusted POR (95% CI)	p-value*
Smoking (yes)	10.18 (11)	10.11 (18)	1.01	0.66 (0.26 - 1.68)**	0.984
Smoking (no)	89.82 (97)	89.89 (160)	(0.46 - 2.22)	1.35 (0.24 - 7.69)***	
Elevated total cholesterol	26.85 (29)	19.10 (34)	1.56	2.08 (1.06 - 4.08)**	**0.031**F
Normal total cholesterol	73.15 (79)	80.90 (144)	(0.88 - 2.74)	1.57 (0,66 - 3.71)***	
Elevated LDL cholesterol	21.29 (23)	12.92 (23)	1.82	2.81 (1.30 - 6.05)**	**0.007**F
Normal LDL cholesterol	78.71 (85)	87.08 (155)	(0.97 - 3.44)	2.46 (0.83 - 7.24)***	
Low HDL cholesterol	56.48 (61)	56.18 (100)	1.01	1.77 (0.67 - 4.66)**	0.960
Normal HDL cholesterol	43.52 (47)	43.82 (78)	(0.63 - 1.64)	1.15 (0.55 - 2.39)***	
Elevated triglycerides	37.96 (41)	32.02 (57)	1.30	1.31 (0.53 - 3.26)**	0.305
Normal triglycerides	62.04 (67)	67.98 (121)	(0.79 - 2.14)	1.24 (0.57 - 2.71)***	
Elevated fasting blood glucose	12.96 (14)	7.87 (14)	1.75	2.79 (1.07 - 7.30)**	**0.030**F
Normal fasting blood glucose	87.04 (94)	92.13 (164)	(0.80 - 3.82)	0.78 (0.30 - 2.06)***	
Elevated HbA1c	8.33 (9)	2.81 (5)	3.14	4.63 (1.16 - 18.49)**	**0.036**
Normal HbA1c	91.67 (99)	97.19 (173)	(1.03 - 9.65)	2.48 (0.49 - 12.47)***	
Elevated BMI	62.04 (67)	59.55 (106)	1.11	2.10 (0.84 - 5.24)**	0.677
Normal BMI	37.96 (41)	40.45 (72)	(0.68 - 1.81)	1.75 (0.84 - 3.66)***	
Increased waist circumference	53,70 (58)	44,38 (79)	1.45	3.28 (1.22 - 8.81)**	**0.016** M
Normal waist circumference	46.30 (50)	55.62 (99)	(0.90 - 2.35)	2.43 (1.12 - 5.27)***	**0.023** OA

* Chi-square test of homogeneity; p<0.05 in bold. ** Adjusted for sex (Mantel–Haenszel), reference category: male. *** Adjusted for age (Mantel–Haenszel), reference category: older adult. HTN: Hypertension; POR: prevalence odds ratio; LDL: low-density lipoprotein; HDL: high-density lipoprotein; HbA1c: glycated hemoglobin; BMI: body mass index; F: female; M: male; OA: older adult.

## Discussion

The results are similar in some cases and differ in others compared with those reported in previous studies. The prevalence of hypertension was lower than that reported in a German population, where it reaches up to 60% as the population ages^2^, and was close to that observed in Tibet (31.40%)^3^. Regarding risk factors, the combined prevalence of overweight/obesity in a Tibetan population was 47.90%^3^, lower than that found in this study; in contrast, in the United States, one study reports figures ranging from 65% to 75% for these conditions^10^. Elevated HbA1c was associated with nearly a threefold increased risk of hypertension (OR: 2.53) in a Sudanese population^6^, slightly lower than that observed in the present study. Hypercholesterolemia due to increased total cholesterol and LDL cholesterol showed similar results in an Algerian population, with a risk of 2.03 for hypertension^14^. For elevated fasting blood glucose, the risk was 1.14-fold^15^, as was increased waist circumference, with a risk ranging from 1.16 to 1.29 in Chinese adults^8^; both were lower than those found in this study. Variations in hypertension frequency and in the association between risk factors and hypertension across studies may be explained by the specific characteristics of each setting and by variations in social determinants of health.

Hypertension affects one in three participants in this study, a proportion that is consistent with global estimates and should prompt targeted action by social stakeholders, given the organ damage this condition causes (particularly to the heart and arteries) and the subsequent cardiovascular diseases, complications, and functional limitations among affected individuals^1^,^16^. As a silent disease, it develops gradually, and at the time of diagnosis, it is often already in advanced stages with established organ involvement. However, early signs of disease onset can be identified promptly to guide preventive and health promotion actions, particularly regarding associated risk factors^17^.

An important risk factor for hypertension is dyslipidemia, characterized by low HDL cholesterol and elevated total cholesterol (adjusted POR=2.08 in women; p=0.031), LDL cholesterol (adjusted POR=2.81 in women; p=0.007), and triglycerides, given its association with cardiovascular disease, atherosclerosis, and stroke^18^. The link between these conditions lies in the fact that dyslipidemia causes vascular endothelial damage and indirectly affects arterial elasticity through impaired endothelial function, primarily driven by elevated LDL cholesterol levels^19^. Maintaining appropriate serum balance among the different components of the lipid profile contributes to proper vascular function.

In addition, increased waist circumference was strongly associated with hypertension (adjusted POR=3.28 in men; p=0.016; adjusted POR=2.43 in older adults; p=0.023), particularly among men. This marker of central obesity appears to be more predictive of cardiovascular risk than BMI, which did not show a significant association in this study. The underlying mechanism of this association is nutritional imbalance, specifically excessive caloric intake that the body does not utilize and instead stores as adipose tissue. This process is further reinforced by low levels of physical activity, sedentary behavior, and regular alcohol consumption, factors that interact synergistically, leading to more severe effects over the course of hypertension^3^,^8^,^9^,^20^,^21^.

HbA1c (adjusted POR=4.63 in women; p=0.036) is a risk factor for both hypertension and diabetes; thus, elevated levels may indicate a link between these conditions in the same patient. This represents a high-risk combination, as both are chronic diseases that affect major target organs, including the brain, heart, and kidneys, and therefore require timely, appropriate, and effective control to prevent physiological damage^6^,^22^,^23^,^24^. Similarly, elevated fasting blood glucose (adjusted POR=2.79 in women; p=0.030) also acts as a risk factor for hypertension. In contrast to HbA1c, it serves as a short-term indicator of plasma glucose fluctuations and contributes to hypertension by altering blood viscosity, impairing normal blood flow, and increasing vascular workload to ensure adequate blood distribution throughout the body^7^,^25^. In contrast, factors that did not show a significant association with hypertension were smoking, HDL cholesterol, triglycerides, and BMI.

In this context, reversal of hypertension risk factors can be achieved through nonpharmacologic, restrictive, and health-promoting interventions, such as reducing or eliminating dietary sodium, which has an antihypertensive effect and markedly reduces the risk of cardiovascular disease. These effects are complemented by increasing intake of vegetables, fresh fruits, fish, cereals, unsaturated fatty acids, and dietary fiber, which also contribute to weight loss and reduced blood pressure. Likewise, regular physical activity provides additional benefits by promoting adequate glucose metabolism, improving blood flow, and maintaining normal blood pressure. Control of alcohol intake, tobacco use, stress, and depression further complements these measures to prevent the onset or progression of hypertension^26^,^27^.

Health education is the most appropriate tool for informing and raising awareness among vulnerable individuals and their families about restrictive and health-promoting measures that help reduce exposure to risk factors, control blood pressure, and support adherence to healthy lifestyles, thereby restoring optimal health status and enabling individuals to enjoy a better quality of life, maintain autonomy, and engage in daily activities. Therefore, it is the responsibility of primary health care professionals to provide the necessary resources to vulnerable populations to implement actions that achieve these objectives^28^.

Although the retrospective nature of data collection may represent a limitation of the study, this is mitigated by the inclusion of the entire population and the use of data from the health center’s official records

## Conclusions

More than one-third of patients had hypertension; most were older adults and women, with an average disease duration of 5 years. The most frequent risk factors were low HDL cholesterol and increased BMI. Elevated HbA1c was associated with more than a threefold increased risk of hypertension. Elevated total cholesterol, LDL cholesterol, and fasting blood glucose were associated with a more than twofold increased risk in women. In men, increased waist circumference was associated with a more than threefold increased risk, and in older adults, with a more than twofold increased risk of the disease.

The risk factors identified in this study are modifiable, and their alteration is primarily attributable to unhealthy dietary patterns and/or insufficient physical activity among patients. Therefore, education on disease management, recommended care practices, and lifestyle modifications should be initiated promptly and prioritized upon diagnosis to ensure an adequate quality of life for affected individuals. These interventions should be implemented through shared agreements among health care professionals, patients, and primary caregivers in order to achieve the proposed goals.
